# Size always matters, shape matters only for the big: potential optical effects of silica bodies in *Selaginella*

**DOI:** 10.1098/rsif.2022.0204

**Published:** 2022-07-06

**Authors:** Ming-Chih Shih, Pei-Jung Xie, Jiannyeu Chen, Peter Chesson, Chiou-Rong Sheue

**Affiliations:** ^1^ Department of Physics, National Chung Hsing University, 145 Xing Da Road, Taichung 402, Taiwan; ^2^ i-Center for Advanced Science and Technology, National Chung Hsing University, 145 Xing Da Road, Taichung 402, Taiwan; ^3^ Department of Life Sciences and Center of Global Change Biology, National Chung Hsing University, 145 Xing Da Road, Taichung 402, Taiwan; ^4^ Department of Ecology and Evolutionary Biology, The University of Arizona, Tucson, AZ 85721, USA

**Keywords:** diffraction lens, papillae, conical, giant chloroplast, wavelength-dependent dispersion, photodamage

## Abstract

Silica bodies are commonly found in *Selaginella*, but their function is unclear. Lens-like appearance and location in many species above giant chloroplasts of dorsal epidermal cells suggest optical functions. Silica body morphology in three *Selaginella* species was studied by microscopy. Optical effects were assessed by wave-optic simulations. Large convex, approximately hemispherical (papillose) and small approximately conical (concave–convex) silica bodies were found in different species. Both types lead to a concentrated spot of light high in the dorsal epidermal cell. Large convex bodies concentrate light 10–25 times in a shape-dependent manner by refraction, and small silica bodies concentrate light in a shape-insensitive, but wavelength-dependent, manner by diffraction (red light: approx. 2.3 times; blue light: approx. 1.5 times). Due to chloroplast movement, this concentrated light is above the chloroplast under high light, but within it under low light. Beyond the spot of concentration, light is dispersed into the chloroplast. Thin *Selaginella* leaves mean these effects may enhance light capture and minimize photodamage, but other effects such as inhibition of herbivory, mechanical support, and immune responses need to be considered. Silica bodies undoubtedly have optical effects, but their significance to the functioning of the plant requires direct studies of ecophysiological performance.

## 1. Introduction

Silica bodies or phytoliths are one type of plant biomineral of amorphous silica (SiO_2_ · *n*H_2_O). Silica mineralization has been a feature of land plant biochemistry for over 400 Myr [1]. Silica bodies are deposited from silicon (Si), which is absorbed by plant roots in the form of silicic acid, and becomes highly polymerized into discrete units [2]. Polymerized silica is one of the hardest materials in the plant tissue, and greatly hardens cell walls [3].

Some benefits of silica bodies in plants have been reported and hypothesized, including mechanical support [4], reducing damage from herbivores and fungi [5–7], and facilitating light harvesting [8–10]. Because silica content in wetland plants negatively correlates with lignin and cellulose, it has been concluded that silica in these plants may replace the mechanical role of these organic polymers [11]. Grasses accumulate much more silica (over 400%) in their leaves after herbivore attack [12]. It was assumed traditionally that silica creates a physical barrier that prevents fungal penetration, but recent studies have suggested that Si acts as a biochemical mediator, because the initiation of the hypersensitive reaction to the fungal attack is faster and more effective in the presence of silicic acid [3].

As the composition of silica bodies is similar to that of transparent glass, they inevitably affect the passage of light into the plant. The window hypothesis postulates that silica bodies facilitate light transmission and thus enhance light harvesting [8–10]. Although a test of this idea in rice did not show the predicted effects on photosynthetically active radiation or improvements in photosynthetic performance [13], silica bodies and Si content have, however, been correlated with reflectance and transmittance spectra in grasses and sedges [14]. However, silica bodies are only one of many leaf surface structures (prickle hair, cuticle and epidermis), along with chlorophyll *a* content and leaf age, that significantly affect spectra [14].

Theoretical analyses have sought to refine the potential optical effects that silica bodies might have on plants. Simulations have implied that different sizes and distributions of silica bodies can importantly influence how light is distributed through a leaf [15]. Although studies of silica bodies have largely focused on angiosperms, silica bodies are found in Selaginellaceae, a structurally different group of plants potentially having different roles for silica bodies. Silica bodies can occur in Sellaginellaceae in high density above chloroplasts in the epidermal cells [16,17], in contrast with angiosperms which never have fully photosynthetic epidermal cell chloroplasts [18].

The monotypic genus *Selaginella*, Selaginellaceae (Lycopodiophyta), one of the most basal vascular plants [19], appears in the fossil record some 200 million years before the appearance of angiosperms. *Selaginella* are found to have the highest Si content (0.69 to 11.32%, dry weight) among the early land plants [1], which is even higher than that of some grasses and sedges [1,20]. Moreover, diverse forms and distribution patterns of silica bodies have been found in *Selaginella* [16,17,21–23]. Uniquely among vascular plants, some shade-adapted *Selaginella* have silica bodies above single giant chloroplasts in the leaf dorsal epidermal cells [23], with unknown consequences. Moreover, some deep shade *Selaginella* have a special giant chloroplast, the bizonoplast (Bp) with dimorphic ultrastructure with potential optical effects influencing photosynthesis [18,24] including iridescence under some circumstances [25].

These *Selaginella* contrast strongly with most land plants, which have multiple small chloroplasts in leaf mesophyll cells [18]. However, the leaves in *Selaginella* are very small, termed microphylls, and their dorsal epidermal cells are the main location of photosynthesis in many species. Thus, the optical properties of epidermal silica bodies have the potential for important effects on light harvesting. It is notable that the silica bodies in *Selaginella* are generally small, approximately 0.5–2 µm [23], compared with silica bodies in grasses up to 50 µm (figures in [26]). Small size means that optical effects of silica bodies in *Selaginella* may be localized on the cellular scale, affecting light passage into the cells most responsible for photosynthesis, the dorsal epidermal cells.

We characterize the silica bodies of three shade-adapted *Selaginella* species with giant chloroplasts in the dorsal epidermal cells. The scale of a silica body makes direct study of the passage of light through the body very difficult. Fortunately, the physics of light optics in such structures can be characterized well by modelling. We present simulations of wave optics based on the Huygens–Fresnel (HF) principle to predict the optical effects of silica bodies on these three species. We first classify the geometry of silica bodies with regard to optical properties, as can be determined by ray tracing, and hence the effects of refraction. However, if the height of a silica body is comparable to the wavelength, effects of diffraction can dominate over refraction. We characterize the predicted effects of silica bodies on the light distribution inside an epidermal cell for the study species and consider the adaptive potential of silica bodies in these *Selaginella*.

## 2. Material and methods

### 2.1. Materials

**Figure 1.**
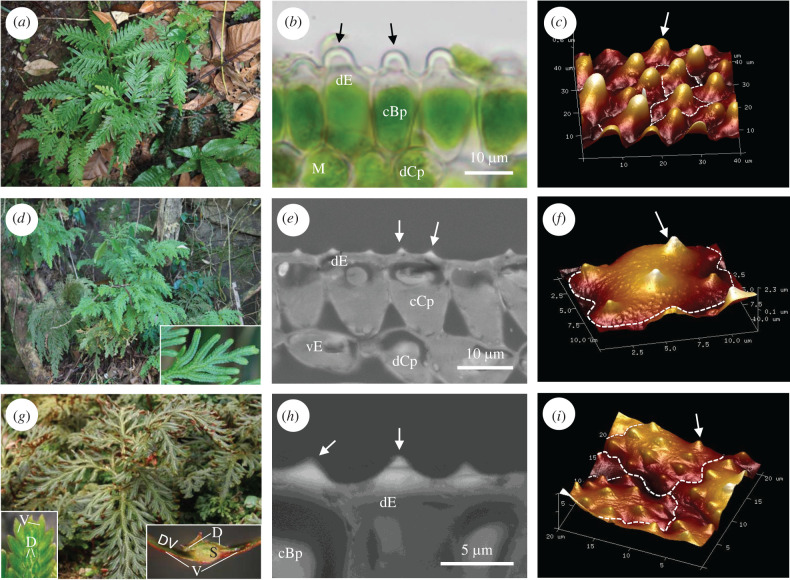
Types of silica bodies on the dorsal side of ventral leaves in three *Selaginella* species. First column: habitats and plant morphology. Second column: transverse leaf structures, free-hand section in (*b*) and scanned resin semi-thin sections in (*e*) and (*h*). Third column: reconstructed three-dimensional AFM images with some cell outlines marked. (*a–c*) *S. delicatula* with papillose silica bodies (arrows) and bizonoplasts. (*d–f*) *S. moellendorffii* with conical silica bodies (arrows) and giant chloroplasts (not bizonoplasts) with typical ultrastructure. (*g–i*) *S. erythropus* with conical silica bodies (arrows) and bizonoplasts. Insets in (*g*) show leaf morphology and a transverse view of a shoot. *Abbreviations:* cBp, cup-shaped bizonoplast; cCp, cup-shaped chloroplast; D, dorsal leaves; dCp, disc-like chloroplast; dE, dorsal epidermal cell; DV, dorsal side of a ventral leaf; M, mesophyll cell; S, stem; vE, ventral epidermal cell; V, ventral leaves.

Three species of *Selaginella* with different types of silica body were chosen (*S. delicatula* (Desv.) Alston, *S. erythropus* (Mart.) Spring and *S. moellendorffii* Hieron.) (figure 1). *Selaginella delicatula* (with Bp) and *S. moellendorffii* (giant chloroplast, without Bp) are commonly found at forest edges or near stream banks at low elevation in Taiwan [27]. *Selaginella erythropus* is a deep-shape plant native to Central and South America [28,29], with Bp (see [30] for details of its cultivation in the laboratory). The two Taiwan *Selaginella* were selected and sampled from 3 to 5 natural populations (table 1). Three individuals were sampled from each population. The size, shape and type of silica bodies on the dorsal surface of ventral leaves were studied. At least 30 silica bodies from each individual were used in the morphometric analysis.

**Table 1.** Morphometric data of silica bodies on the dorsal epidermal cells of three species of *Selginella*. Continuous measurements are mean ± s.e.

character	*S. delicatula*^d^	*S. erythropus*^e^	*S. moellendorffii*^f^
morphology and no. per cell	1 papillose	5–13 small conical^a^	5–10 small conical^b^
average height (μm, *h*_Si_)	3.44 ± 0.02	1.12 ± 0.01	1.27 ± 0.02
average base diameter (μm, *d*_base_)	6.49 ± 0.03	2.12 ± 0.03	2.71 ± 0.04
average aspect ratio (*r*_asp_, *h*_Si_/*d*_base_)	0.53 ± 0.003	0.53 ± 0.004	0.47 ± 0.004
average cell coverage for cells with silica bodies (%)	19.02 ± 1.12	23.01 ± 1.48	13.75 ± 0.72
whole-leaf coverage (includes cells without silica bodies) (%)	16.84 ± 2.13	6.42 ± 1.15	3.71 ± 0.49
light dispersion by diffraction^c^ at 450 nm	4.8^◦^	14.9^◦^	13.1°
light dispersion by diffraction^c^ at 650 nm	6.9^◦^	21.5^◦^	18.9^◦^

^a^[23].

^b^[39].

^c^Calculations in the electronic supplementary material.

^d^Samples from Taipei areas (Xianjiyan Trail, Manyueyuan National Forest Recreation Area) and Central Taiwan (Fairy Waterfall, Guanyin Waterfall).

^e^Laboratory cultivation as in [30].

^f^Samples from New Taipei City (Manyueyuan National Forest Recreation Area), Hsinchu (Cinsbus) and Pingtung (Liangshan Waterfall).

### 2.2. Topography and distribution of silica bodies

The shapes, types and distribution patterns of silica bodies on dorsal surfaces of ventral leaves were identified by a tabletop scanning electron microscope (tSEM, TM3000, Hitachi, Tokyo, Japan). For this technique, cleaned leaves were mounted on a stub with carbon conductive double-sided tape. Three-dimensional images and surface topography were obtained with an atomic force microscope (AFM). For AFM, detached fresh leaves were attached on slides with conductive copper foil double-sided tape and observed with a scanning probe microscope system (Bruker Dimension Icon, Bruker BioSpin Corporation, Billerica, USA). The NCSTR series AFM probe (Nanoworld Co., Neuchâtel, Switzerland) was operated under tapping mode with a scan rate of 0.3–0.6 Hz, scanning through 5–15 µm^2^ at a resolution of 512 by 256 pixels (see details in [23]). The three-dimensional images of silica bodies were characterized with NanoScope Analysis software (v. 7.4).

### 2.3. Sectioning

For free-hand sections, the middle part of a fresh leaf was cut with a razor blade under a stereo microscope (Leica S8 APO, Leica Microsystems, Wetzlar, Germany). Semi-thin sections were prepared using a standard protocol [24]. Both free-hand and semi-thin sections were observed with a light microscope (Olympus, BX51, Tokyo, Japan) and photographed (EOS 700D, Canon, Tokyo, Japan). For *S. erythropus* and *S. moellendorffii* (with smaller conical silica bodies), the semi-thin sections were further observed under tSEM (TM3000, Hitachi, Tokyo, Japan) to obtain morphometric data at higher magnification. Anatomical features, including height and width, of silica bodies were measured manually in ImageJ (v. 1.52t, National Institutes of Health, USA).

### 2.4. Classification of silica body geometry for optical property studies

**Figure 2.**
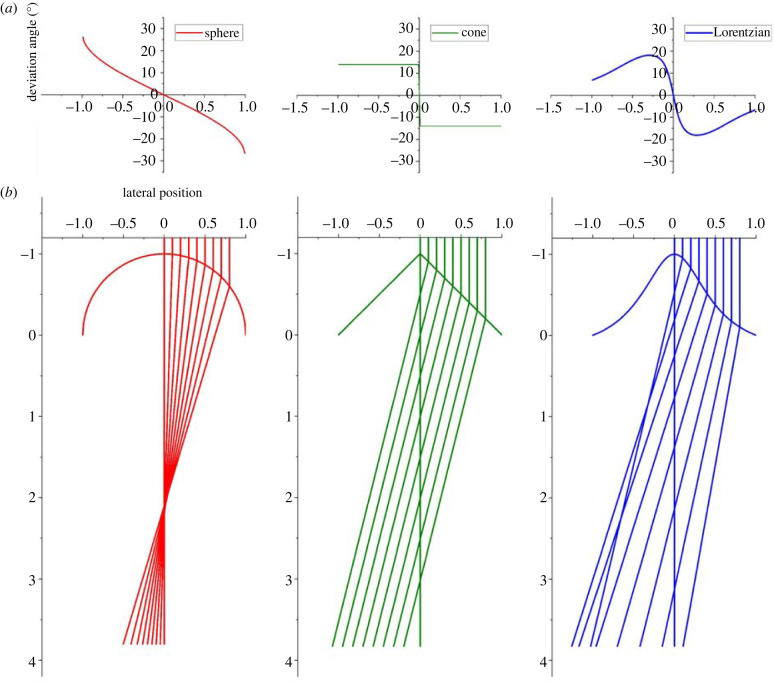
Transmitted light beam behaviour for silica body classification. (*a*) Deviation angles are plotted as functions of the lateral position, with 0 below the apex (arbitrary units). Based on Snell's law, the absolute angles, as functions of the absolute value of the lateral position, are monotonically increasing (convex, red), a step function (cone, green) or hump-shaped (concave–convex shape, blue) reaching a maximum at the point of inflection of the concave–convex curve. (*b*) Ray tracing diagram demonstrating converging (red), parallel (green) and diverging (blue) transmitted light beams as normal incident light passes through convex, conical and concave–convex interfaces, respectively. Note that the outcome for the left side of a silica body is merely a reflection of the right side, but for clarity is not drawn.

To characterize the shape of a silica body relevant to light refraction (figure 2), we use the predictions of Snell's law. Incident light propagating across an interface will deviate according to the change in refractive index. From Snell's law, we can calculate *θ*_dev_ (the difference between the incoming and outgoing angles) via the formula

2.1 $$\theta_{\mathrm{dev}}=\theta_{\mathrm{inc}}-\sin^{-1}\left(\frac{\sin\theta_{\mathrm{inc}}}{n}\right),$$

where *θ*_inc_ is the angle between the incident light and the normal to the surface and *n* is the relative refractive index $\equiv n_{\mathrm{silica}\;\mathrm{body}}/n_{\mathrm{air}}$. We assume *n*_air_ is adequately approximated by 1. Figure 2*b* shows the simulated ray tracing for parallel vertical light rays propagating across different interfaces: a cone (in parallel), a sphere (convergently) and a Lorentzian curve (divergently). Figure 2*a* plots *θ*_dev_ from equation (2.1) as a function of the lateral position of a vertical light ray. The cone shape gives a constant *θ*_dev_ due to a constant surface slope, but a sphere (convex) exhibits monotonically decreasing *θ*_dev_ due to its constant curvature. The Lorentzian (concave–convex), initially shows a sharp decrease in *θ*_dev_ reaching a minimum for a lateral deviation through the inflection point of the Lorentzian curve, after which the surface is concave and *θ*_dev_ increases towards 0. Mathematical functions (electronic supplementary material, S1–S4) were fitted to the digitized profile data from microscopy using OriginPro (2020, Origin Lab, USA). For each case, a function with a good fit to the data, judged by the squared deviation, was selected.

In our simulations, we simplified the calculation by approximating all silica bodies as rotationally symmetric around a central *z*-axis normal to the surface. With normal incident light, crossing points on the central axis have higher intensity due to merging of light from concentric rings on the surface of the silica body. The distribution of this merged light on the central axis can be related to the silica body profile as a convergent depth profile (convex, focal-like pattern) or a divergent depth profile (concave, continuous spread). A concave–convex curve is characterized by its inflection point which determines the boundary between the concave and convex regions of the surface and corresponds to a reversal of the sign of the change in slope with lateral position. This slope reversal defines the onset of the divergent beams. The location of the inflection point greatly affects the light distribution. When it is close to the apex of the silica body, the light is widely diffused because most light strikes a concave surface. However, if the inflection point occurs near the base, then most of the silica body has a convex surface and the light distribution therefore resembles that from a purely convex surface.

### 2.5. Simulation with propagating waves

For wave simulations, five functional curves were selected to capture the range of optical effects to be expected from silica bodies: triangular cones with flat sides, spheres and ellipsoids (convex ideal focal surfaces), and Gaussian and Lorentzian function surfaces with inflection points as concave–convex surfaces. Although each of the selected shapes have their own mathematical parameters, such as apex angle, radius, eccentricity and peak width, a unified treatment requires defining a single parameter to describe relative height and width dimensions. The obvious, easily measured quantity is the aspect ratio, *r*_asp_, defined as the silica body height over its basal diameter (*h*_Si_*/d*_base_). In our simulations, *r*_asp_ was used to vary the fraction of a given fitted function above the cell surface and thus the optical properties for each of the modelled geometries (electronic supplementary material, S1 and S2).

We simulated the optical properties of silica bodies with normal plane wave incident light beams according to the HF principle [31]. Light propagates as an electromagnetic wave with intensity proportional to the square of the propagating electric field (E field). The HF principle describes the propagating wavefront as a linear superposition of waves from a collection *S* of point sources. Thus, the E field, *E*(*P*), at a particular point *P* is the integral over *S*:

2.2 $$E(P)=A{\int \int }_{S}^{}e^{ik_{o}z(s)}\frac{e^{ik\cdot r}}{r}\Theta (\theta )\;ds,$$

where *s* is a point in *S*, ***k*** is the light propagation direction (*k*_0_ = |k|=2π/λ, with *λ* being wavelength in air), ***r*** is the direction *s* to *P* (*r* = |***r***|), *z*(*s*) ≡ *h*_Si_ – *h*(*s*) is the difference between the maximum silica body height *h*_Si_ and the height of surface *h*(*s*) at *s*, and *Θ*(*θ*) is the inclination factor accounting for the spherical wavelet amplitude variation with the angle of direction *θ*. The refractive index, *n*, in the silica body enters as |k|=2πn/λ for ***k*** as a function of *s* in the integral. The product$\;k_{o}z(s)$ carries phase information describing the geometry of the surface. The function *z*(*s*) describes the surface profile; thus, different topographies have different functional forms (electronic supplementary material, S2).

Our simulations do not take account of the interior structure of a cell, which includes numerous organelles. The light path is sensitive to every organelle visible without staining in fresh material or with a refractive index significantly different from that of water. In our materials, only chloroplasts seem likely to have much effect. Moreover, a large vacuole with a homogeneous aqueous interior and ideal optical properties is often above the chloroplast [18]. Thus, in our materials, the complications of cellular organelles other than the chloroplast seem unlikely to affect our results.

## 3. Results

### 3.1. Morphology of silica bodies

Microscope images reveal the morphology of silica bodies on the dorsal epidermal cells of ventral leaves in three *Selaginella* species (figure 1). Morphometric data are given in table 1. Two types of silica body were found. *Selaginella delicatula* has papillose silica bodies, convex in shape, while the other two species (*S. erythropus* and *S. moellendorffii*) have small approximately conical (tending to concave–convex) silica bodies. Although in *S. delicatula* there is only one silica body per dorsal epidermal cell, in the other two species, there are 5–13 silica bodies on dorsal epidermal cells where they are present. In *S*. *delicatula*, silica bodies are found on most dorsal epidermal cells, but in *S. erythropus* and *S. moellendorffii*, many dorsal epidermal cells do not have silica bodies, including those cells in locations shaded by other leaves. Thus, in these two species, whole-leaf coverage by silica bodies is much less than the average coverage on a cell with silica bodies (table 1).

### 3.2. Prediction of focal position in the ray optics regime

**Figure 3.**
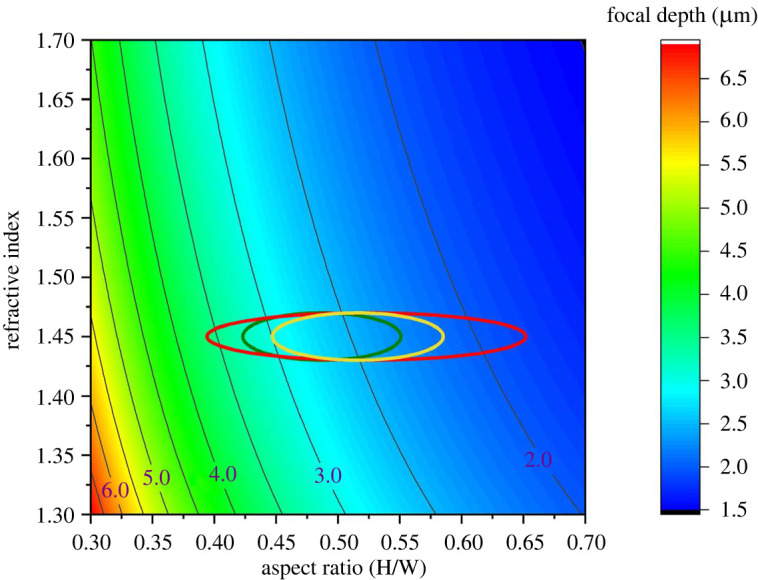
Chart for quick estimation of the light convergence position scaled with height, *f’* ≡ *f/h*_Si_, for parabolic surfaces as a function of the aspect ratio and refractive index. Circled ranges are estimated *f’* values for *S. moellendorffii* (green), *S. erythropus* (red) and *S. delicatula* (yellow).

To accommodate simulation results for size variation in silica bodies, we present simulation calculations, including wavelength, scaled relative to the height *h*_Si_ of the silica body. Therefore, instead of focal length, *f*, we can predict the dimensionless focal length ${\;f}^{\prime }\equiv f/h_{Si}\;$ as a function of the aspect ratio *r*_asp_ and refractive index *n* for convex silica bodies (figure 3; electronic supplementary material, S3). We use this plot for approximate determination of the range of potential focal positions with measured silica body *r*_asp_ and estimated *n* in the ray optics limit. However, unless the convex surface is an ideal focal surface, light will not necessarily converge to a single point, but the error is small for small aspect ratios (electronic supplementary material, S3).

### 3.3. Simulation with propagating waves I: refraction effects

**Figure 4.**
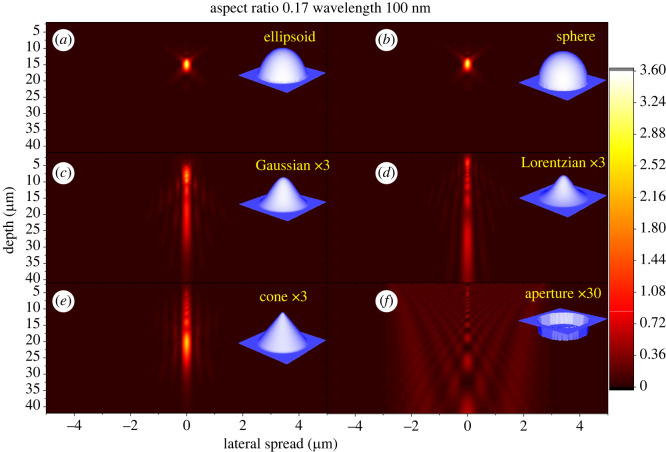
Light distribution patterns for various interfaces (100 nm wavelength). (*a,b*) Convex silica body surfaces exhibiting focus-like convergence spot patterns. (*c,d*) Concave–convex surfaces showing band-like patterns. (*e*) A conical surface and (*f*) a flat interface (aperture like) are included for reference. All structures depicted have the same *r*_asp_ = 0.17 and height *h*_Si_ = 1. Depth = distance below the cell surface. Colour bar: intensity relative to incident light intensity.

**Figure 5.**
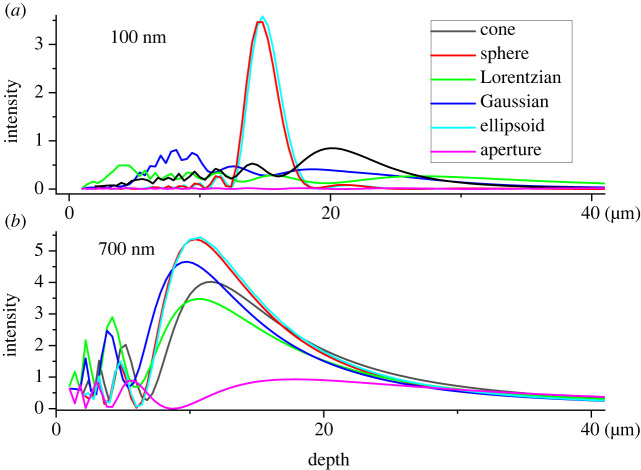
Light intensity distribution along the central axis of a silica body at wavelengths (*a*) 100 nm and (*b*) 700 nm for various interfaces. All interfaces, *r*_asp_ = 0.17 and height *h*_Si_ = 1.

Wave simulations in the case of wavelengths that are short relative to silica body height (figure 4; *d*_base_ : *h*_Si_ : *λ* = 5.88 : 1 : 0.1) give results anticipated by ray optics (figure 2). Differences in light patterns are distinctive: a focal spot pattern for convex surfaces versus a central bright band for concave–convex ones. Intensity of these patterns along the central axis is plotted in figure 5*a*. Spherical and ellipsoidal shapes exhibit peak intensity close to the predicted focal spot, but peak intensity is about 0.2 µm shorter for the ellipsoid, as expected. Both Gaussian and Lorentzian shapes do not create an obvious bright spot (figure 4*c,d*), but their light intensity patterns (figure 5*a*) decay much more slowly than those of convex shapes, leading to visible light bands on the central axis. The triangular cone with flat side walls naturally leads to a pattern intermediate between those of convex and concave–convex surfaces. However, a bright spot is still evident (figure 4*e*), but is attenuated vertically (figure 5*a*), principally below the bright spot. A maximum intensity spot is still clearly evident near the corresponding focal point of the spherical and ellipsoidal shapes.

### 3.4. Simulation with propagating waves II: diffraction effects

**Figure 6.**
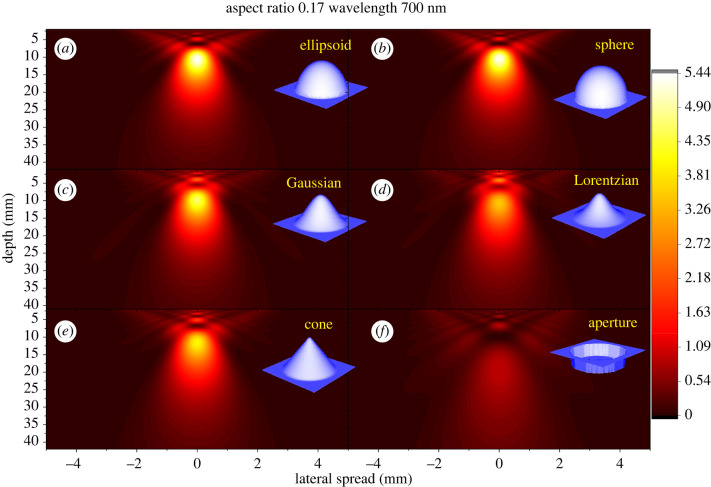
Light distribution patterns for various interfaces (700 nm wavelength). Unlike 100 nm, all patterns are similar showing high-intensity spots of different sizes depending on the interface. (*a,b*) Convex silica body surfaces. (*c,d*) Concave–convex surfaces. (*e*) A conical surface and (*f*) a flat interface (aperture like) are included for reference. All structures depicted have the same *r*_asp_ = 0.17 and height *h*_Si_ = 1. Depth = distance below the cell surface. Colour bar: intensity relative to incident light intensity.

When the silica body size is comparable to the wavelength (*d*_base_/*λ ∼* 10^0^), diffraction effects become non-negligible and so interference will occur, which cannot be accounted for in ray optics (figure 5*b*). Figure 6*a–f* shows simulation results for long wavelengths (*d*_base_ : *h*_Si_ : *λ* = 5.88 : 1 : 0.7), in the same geometric structures investigated in the previous section. Long-wavelength simulations give dramatically different patterns compared with the short-wavelength simulations shown in figure 4. Most strikingly, figure 6 shows that the difference between convex and concave–convex surfaces is smeared out by diffraction. All geometries exhibit similar single blur peak distribution patterns with only minor distinctions in intensities and peak widths. To understand how much of the pattern is due silica bodies rather than simply windows on the cells, the pattern originating from a flat circular aperture of the same basal diameter was simulated (figure 6*f*). This comparison reveals that the extrusive silica bodies, whether convex or concave, do amplify the overall intensity around one point into a clear bright spot.

**Figure 7.**
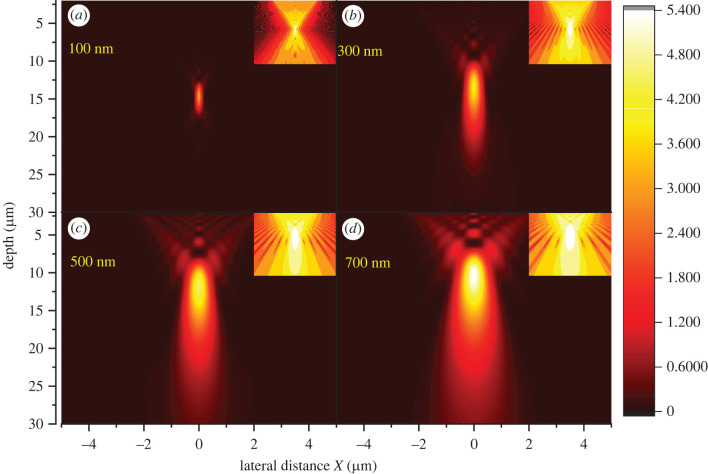
Appearance of interference effects as wavelengths increase. The contribution of interference effects increases with the wavelength, causing the intensity pattern to change. (*a*) 100 nm, (*b*) 300 nm, (*c*) 500 nm and (*d*) 700 nm. Insets show intensity on a log scale. Simulation for a spherical surface with *r*_asp_ = 0.17 and height *h*_Si_ = 1.

**Figure 8.**
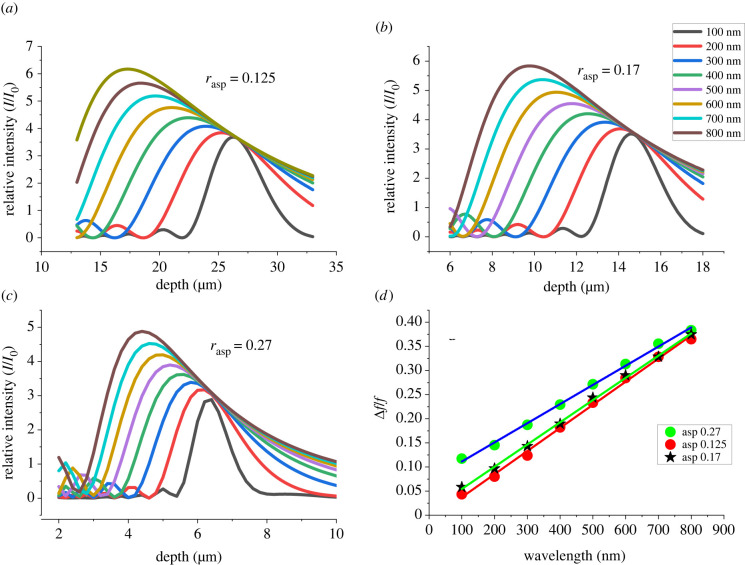
Change in the position of the light intensity maximum with wavelength and aspect ratio for convex silica bodies. (*a–c*) Intensity patterns with depth for a range of wavelengths. For each wavelength, a single strong maximum is present, which increases in height and moves to the left (nearer the base of the silica body) as the wavelength is increased. The patterns are on different scales for different aspect ratios, but (*d*) shows that these changes can be understood from comparison with the change in the focal position relative to that predicted by ray optics (Δ*f*/*f*_0_) where similar linear relationships are shown for different aspect ratios.

Figure 7*a–d* shows how interference gradually alters the ray optic pattern into a diffraction-dominated pattern with increasing wavelength. Increase in the spot width as the wavelength increases is typical of diffraction. It occurs because the phase difference resulting from the optical path length difference is in inverse proportion to the wavelength. However, the maximum intensity also gradually increases and the peak position moves up towards the cell surface at longer wavelengths (figure 8*a–d*). This peak shifting is essentially a form of chromatic aberration induced by diffraction, not to be confused with conventional chromatic aberration from refraction, which is much smaller (electronic supplementary material, S5).

### 3.5. Simulation of silica bodies from three *Selaginella* species

To apply these ideas to the silica bodies of *Selaginella*, two wavelengths representing chlorophyll's major absorptions in blue (450 nm) and red (650 nm) regions of the spectrum were selected for simulations. Geometric data describing typical silica bodies of *S. delicatula*, *S. erythropus* and *S. moellendorffii* were chosen as average values from the SEM and AFM measurements (table 1). The silica body of *S. delicatula* is fitted with a convex shape. The concave–convex silica bodies of *S. erythropus* and *S. moellendorffii* are better described with a Lorentzian function.

**Figure 9.**
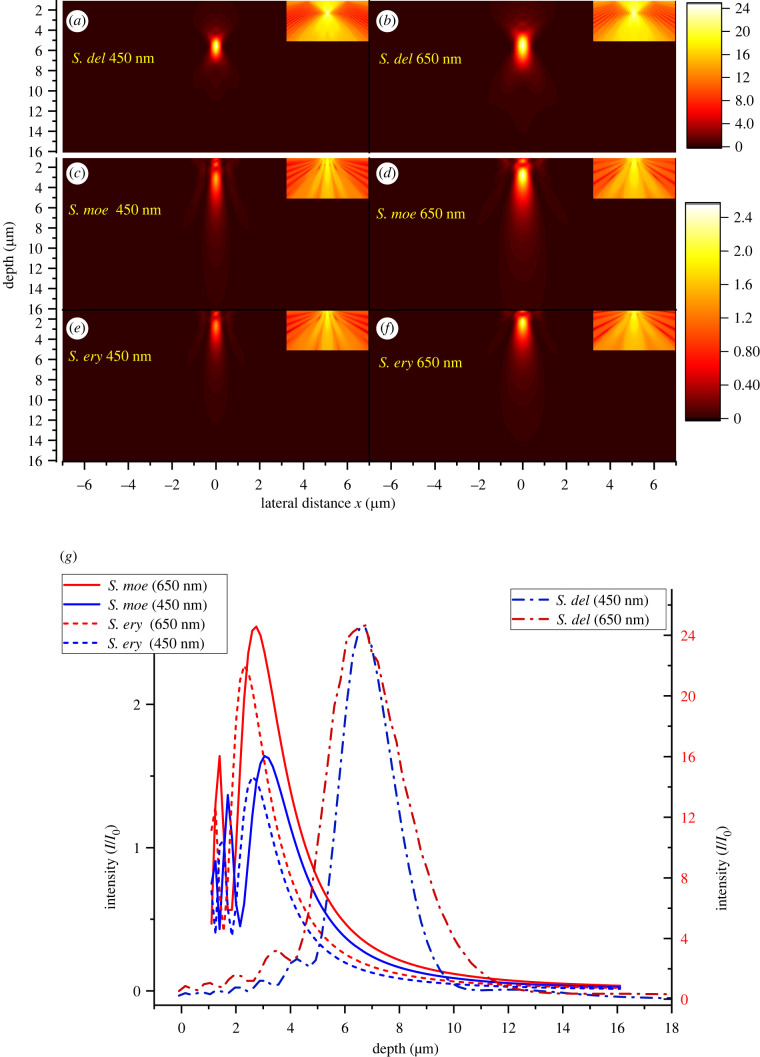
Simulated blue (450 nm) and red (650 nm) light intensity distributions for *Selaginella* silica bodies based on measured dimensions. Insets for (*a–f*) show the distributions on a log scale of intensity. (*a*,*b*) *S. delicatula.* The convex surface profile displays the ray optics focal lens feature. (*c–f*) *S. moellendorffii* and *S. erythropus.* Concave–convex surface profiles. The predicted band structure of ray optics is not evident. Instead, diffraction dominates the simulation and once again shows a focal-like distribution in all cases. (*g*) Intensity profiles along the central axis. The scale difference between *S. delicatula* and the other species reflects the approximately 10-fold basal area difference. Simulations are based on the average dimensions from table 1. Shapes are based on profile fitting.

The large size of the convex *S. delicatula* silica bodies leads to maximum transmitted light peaks, according to Fresnel's propagation waves, close to the depth predicted by ray optics (off by 0.5 µm from equation (3.1)) for both 450 nm and 650 nm wavelengths, as shown in figure 9. This simulation result with an ideal spherical surface indicates that the silica body of *S. delicatula* can function as a focal lens with the intensified region extending about 10 times the wavelength, and peak intensity reaching about 25 times that of incident light, as suggested by figure 9*g*. Although a deviation from this ideal surface would reduce the maximum intensity, the maximum intensity would still be enhanced approximately fourfold even for a concave–convex Lorentzian surface. The ideal spherical result is used as a reference for estimating the potential reduction in the intensification of the bright spot due to non-ideal surfaces.

Although *S. erythropus* and *S. moellendorffii* have silica bodies characterized as concave–convex curves described by a Lorentzian function, their transmitted light distributions did not display the central band predicted by ray optics. This result is due to the small sizes of these silica bodies (table 1), which lead to diffraction-dominated outcomes. In the simulations, both species show clear wavelength-dependent bright spot position shifts between 450 nm and 650 nm of about 13% (*S. erythropus* approx. 0.3 µm; *S. moellendorffii* approx. 0.4 µm). Intensity enhancement is also observed. In *S. erythropus* intensity is enhanced 2.3 times at 650 nm and 1.5 times at 450 nm. Corresponding enhancements for *S. moellendorffii* are 2.5 times and 1.6 times at 650 nm and 450 nm (figure 9*g*). The intensified region (where light intensity is higher than incident light) is longer in *S. delicatula* than in the other two species due to its much higher peak intensity. *Selaginella erythropus* has a shorter intensified region than *S. moellendorffii* (approx. three times wavelengths), which is caused by its larger aspect ratio.

## 4. Discussion

### 4.1. Refraction effects

Studies in various plants species have suggested that silica bodies lead to more effective use of light in photosynthesis by redirecting sunlight towards chloroplasts [15] or scattering the light with the result that the light path inside the leaf is longer with more opportunities for absorption by chloroplasts [15,32]. These strategies work intercellularly but may not be effective in ultrathin leaves, such as those in *Selaginella*. In the species studied, silica bodies are found above the main photosynthetic cells, which are the dorsal epidermal cells. Their sizes and shapes differ between species, but the optical outcomes are similar. In all cases, our studies predict that light normal to the leaf surface would be concentrated by the silica bodies in the upper part of the epidermal cell and would then disperse further into the cell. This outcome, however, is due to a different mechanism in *S. delicatula* compared to the other two species. In *S. delicatula*, the large size and convex shape of the silica body mean that the effects are due to refraction, and can be analysed by ray optics. Refraction leads to a focus, and hence a spot of high light intensity in the upper part of a dorsal epidermal cell.

### 4.2. Diffraction effects

In *S. erythropus* and *S. moellendorffii*, the concave–convex shape does not produce a focus by refraction. Indeed, ray optics predict light dispersion. However, the small size of the silica body, which is comparable to the wavelength, leads to the dominance of wave optics. Passage of light through the centre of the silica body, where it is thickest, increases the optical path length due to the high refractive index of silica (*n* approx. 1.45). This path length matches, to less than the wavelength, the optical path length through thin silica on the periphery. Thus, light from these different paths arrives with a similar phase at one spot where they reinforce to give high light intensity. Notably, silica body shape has very little role in this outcome because the shape variations are minor compared with the relevant wavelengths of light. The technical details are to be found in the electronic supplementary material, S4 and S5. Light disperses beyond the points of concentration in all species. However, the light dispersion due to diffraction (*S. erythropus* and *S. moellendorffii*) increases with the wavelength, and is thus about 50% greater for red light in comparison with blue light (table 1). By contrast, the dispersion effects for *S. delicatula* are much less affected by diffraction.

### 4.3. Effects of chloroplast movement

Light intensity can vary greatly within a habitat and over time. Under the higher intensities experienced by these *Selaginella*, e.g. during sunflects, the giant chloroplast sits low in the dorsal epidermal cell [33]. The high-intensity spot predicted by our calculations would occur above the giant chloroplast in all species under conditions of higher incident light. However, due to chloroplast movement [33–35], this high-intensity spot would occur in the upper part of the chloroplast at the lower levels of incident light. These changes are potentially advantageous because under higher incident light, the high-intensity spot might cause photodamage if it were to occur within the chloroplast [36], or inefficient photosynthesis due to saturation of the light response curve [30]. Instead, under these higher light conditions, the chloroplast would intercept the more dispersed light beyond this spot. With lower light, the location of the high-intensity spot within the chloroplast should not have such disadvantages and should increase the light intercepted by the giant chloroplast.

### 4.4. Passage of light through the cell

The very thin leaves of these shade-adapted *Selaginella* mean that any photon of light striking a leaf from above has only a few opportunities for absorption by a chloroplast, primarily in the dorsal epidermal cells, although limited small chloroplasts are present in other tissues [24,37,38]. If a photon of light is not absorbed by the dorsal epidermal chloroplast in the cell that it strikes, it may well be lost. However, the dorsal epidermal cells in these and many other shade-adapted *Selaginella* [18] are funnel-shaped (more precisely paraboloid) and surrounded by intercellular air space, as shown most clearly with semi-thin and ultrathin sections [24,30]. Consequently, light striking the cell boundary from the interior of the cell at an obtuse angle would have high probability of being reflected back into the cell (see fig. 7 of [18]). Thus, any given photon of light would have multiple opportunities to be absorbed by the chloroplast. However, light passing vertically through the cell in the central region would strike the cell boundary approximately perpendicularly, and so likely pass out of the cell, precluding further opportunities for absorption by the chloroplast. The optical effects of the silica body would greatly diminish this outcome because most light passing vertically through the silica body would be dispersed, once again creating many opportunities for absorption by the chloroplast.

**Figure 10.**
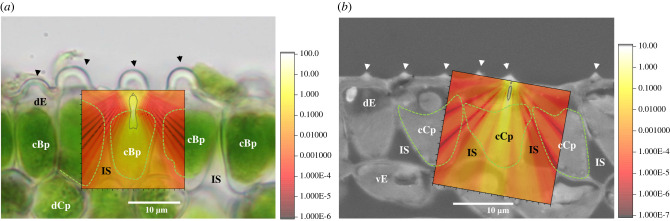
Illustration of intracellular light redistribution after passage through a *Selaginella* silica body. Light distribution simulations are overlaid on images of transverse leaf sections. (*a*) *S. delicatula* and (*b*) *S. moellendorffii.* In both species, the zone of light convergence is located in the upper part of the epidermal cell. Diffuse light spreads to the cell wall, where it may be affected by the air space around the funnel-shaped epidermal cells, as labelled on an adjacent cell. Arrows indicate the locations of silica bodies, dotted lines mark the giant chloroplasts. *Abbreviations*: cBp, cup-shaped bizonoplast; cCp, cup-shaped chloroplast; dCp, disc-like chloroplast; dE, dorsal epidermal cell; IS, intercellular space; vE, ventral epidermal cell.

Light passing vertically into a dorsal epidermal cell need not pass through a silica body, but due to the funnel shape of the cell, passage away from the centre of the cell would strike the interior cell boundary at an obtuse angle and therefore be totally or near totally reflected back into the cell (figure 10). Thus, the combination of the silica body and the cell shape in *S. delicatula*, where the silica body is in the centre of the surface of the dorsal epidermal cell, should lead to multiple opportunities for light absorption regardless of where a photon enters the dorsal surface of the cell. In *S. erythropus* and *S. moellendorffii*, however, several silica bodies are distributed across the dorsal surface of the cell, and what happens to light that does not pass through a silica body depends greatly on the details of the silica body distribution. Should the spacing of two or more silica bodies be comparable to the wavelength, the light passing between these silica bodies would also be subject to dispersion by diffraction. Light passing vertically in other areas without silica bodies would strike the cell boundary at an obtuse angle if in peripheral regions of the cell, as for *S. delicatula*, but light striking in the central region would potentially pass out of the cell in the absence of a silica body if not absorbed first by the chloroplast. Overall, the silica bodies in *S. erythropus* and *S. moellendorffii* have the potential to lead to much light dispersion, creating multiple opportunities for absorption by the chloroplast; but in the absence of studies of the precise chloroplast distribution, we do not have an estimate of how much light would be dispersed or reflected from the cell boundary.

### 4.5. Effects of silica body distribution patterns

Although silica bodies are mostly evenly distributed over the dorsal surfaces of the leaves in *S. delicatula*, this is not the case for *S. erythropus* [23] and *S*. *moellendorffii* [39]. In the latter two species, areas without silica bodies include the parts of ventral leaves shaded by other leaves. Dorsal leaves directly exposed to the sun have typically more abundant silica bodies [23], but even there, the distribution on the leaf tends to be clustered. However, in *S. erythropus*, the ventral sides of ventral leaves (but not ventral sides of dorsal leaves) have abundant silica bodies. These ventral silica bodies are larger in size than the dorsal silica bodies [23], but since they point down, they normally would receive no light from the exterior of the leaf. Instead, they have the potential to reflect some light back into the leaf. By contrast, the ventral sides of ventral leaves of *S. moellendorffii* have silica bodies only along the leaf margin and on each side of the midrib, and the ventral sides of ventral leaves of *S. delicatula* are devoid of silica bodies [38].

### 4.6. Non-optical effects of silica bodies

These observations strongly suggest that the roles of silica bodies may differ depending on species and their locations on the leaves. Moreover, their main advantages to a plant may not be their optical effects. For instance, many other roles of silica bodies have been suggested for plant species including inhibition of herbivory or fungal infection, and mechanical support [40,41]. However, to our knowledge, studies supporting these other roles have not been published for *Selaginella*. A potential optical effect of silica bodies beyond any roles they may have in photosynthesis relates to the immune response in plants. In *Arabidopsis thaliana* (L.) Heynh., it has been reported that a low-light environment speeds the progression of bacterial infection [42]. However, prior exposure to excess light leads to a better response to infection. Importantly, this immune response does not require global excess light exposure. Indeed, local chloroplast exposure will induce a systemic immune response for the whole plant [43]. If applicable to *Selaginella*, this effect on the immune response might still work due to the high light intensity spots caused by silica bodies over just part of a leaf. This effect might aid survival in a low-light environment.

### 4.7. Conclusion

The intriguing silica bodies of *Selaginella* pose serious questions about functional roles. Being located above the main sites of photosynthesis naturally raises the questions of optical effects. We have shown that the silica bodies in the species studied have the potential to concentrate light at one spot in the dorsal epidermal cell. Large convex bodies concentrate light in a shape-dependent manner; however, the concentration of light by small silica bodies is shape-insensitive, but wavelength-dependent. Due to chloroplast movement, this concentrated light is above the chloroplast under high light, but within it under low light. Dispersion of light occurs beyond the spot of concentration into the chloroplast. In conjunction with cell properties and silica body locations, optical effects are created that may enhance light capture and minimize photodamage. A question remains as to whether these optical effects are key adaptations or are minor roles that silica bodies have. Resolution of this issue may need to await detailed physiological and ecological studies of populations with and without silica bodies.

## Data Availability

The data are provided in the electronic supplementary material [44].
